# G2P Provides an Integrative Environment for Multi-model genomic selection analysis to improve genotype-to-phenotype prediction

**DOI:** 10.3389/fpls.2023.1207139

**Published:** 2023-08-04

**Authors:** Qian Wang, Shan Jiang, Tong Li, Zhixu Qiu, Jun Yan, Ran Fu, Chuang Ma, Xiangfeng Wang, Shuqin Jiang, Qian Cheng

**Affiliations:** ^1^ Frontiers Science Center for Molecular Design Breeding, China Agricultural University, Beijing, China; ^2^ National Maize Improvement Center of China, College of Agriculture and Biotechnology, China Agricultural University, Beijing, China; ^3^ Key Laboratory of Biology and Genetics Improvement of Maize in Arid Area of Northwest Region, Ministry of Agriculture, Northwest A&F University, Yangling, Shaanxi, China; ^4^ State Key Laboratory of Crop Stress Biology for Arid Areas, Center of Bioinformatics, College of Life Sciences, Northwest A&F University, Shaanxi, Yangling, China

**Keywords:** genomic selection, genotype-to-phenotype prediction, singularity container, crop breeding, multi-model integration

## Abstract

Genotype-to-phenotype (G2P) prediction has become a mainstream paradigm to facilitate genomic selection (GS)-assisted breeding in the seed industry. Many methods have been introduced for building GS models, but their prediction precision may vary depending on species and specific traits. Therefore, evaluation of multiple models and selection of the appropriate one is crucial to effective GS analysis. Here, we present the G2P container developed for the Singularity platform, which not only contains a library of 16 state-of-the-art GS models and 13 evaluation metrics. G2P works as an integrative environment offering comprehensive, unbiased evaluation analyses of the 16 GS models, which may be run in parallel on high-performance computing clusters. Based on the evaluation outcome, G2P performs auto-ensemble algorithms that not only can automatically select the most precise models but also can integrate prediction results from multiple models. This functionality should further improve the precision of G2P prediction. Another noteworthy function is the refinement design of the training set, in which G2P optimizes the training set based on the genetic diversity analysis of a studied population. Although the training samples in the optimized set are fewer than in the original set, the prediction precision is almost equivalent to that obtained when using the whole set. This functionality is quite useful in practice, as it reduces the cost of phenotyping when constructing training population. The G2P container and source codes are freely accessible at https://g2p-env.github.io/.

## Introduction

Genomic selection (GS) is the process of genomically estimating breeding values based on genotype-to-phenotype (G2P) prediction and was originally utilized in animal breeding for estimating the breeding values of untested individuals by analyzing the genotype of a sample (Meuwissen et al., 2001). Recently, GS has been proposed as a promising approach for crop breeding as the dramatic decrease of genotyping expense (Jannink et al., 2010; Hickey et al., 2019). The core idea of GS is to predict phenotypes from genotypes of breeding individuals, allowing a breeder to select the best genetic material to produce a desired phenotype. Therefore, GS as a revolutionary method of precision breeding offers multiple advantages over traditional breeding: it greatly shortens the breeding cycle; it reduces the cost of phenotyping; and it allows more genetic gains per unit time (Jannink et al., 2010; Hickey et al., 2019; Yan and Wang, 2022a). Recently, successful applications of GS in crop breeding have been reported in a variety of crops such as maize, barley, wheat, rice, soybean and rapeseed (Zhao et al., 2012; Nielsen et al., 2016; Belamkar et al., 2018; Kumar et al., 2019; Wang et al., 2021; Hu et al., 2022).

Despite the great potential of GS-assisted breeding, its broad application in crops has been impeded by the complex situation in crop breeding. For instance, multiple statistical, Bayesian, and machine learning (ML) algorithms have been developed to construct GS models in crops, but prediction precisions may vary when applying different models on different species and traits to certain degrees (Heffner et al., 2011; Ornella et al., 2014; Yan et al., 2021). This is because prediction accuracy is influenced by many factors, including the size of training and testing samples, the genetic structure of the breeding population, the density of whole-genome marks, the heritability of target traits, and the span of linkage disequilibrium (Wray et al., 2013). Therefore, to ensure the effectiveness of GS prediction, it is crucial to select the most appropriate GS model based on a comprehensive evaluation of all the methods using optimized parameters from a model library.

Data as of 2021, many GS packages have been developed, such as “rrBLUP” for ridge regression best linear unbiased predictor model (RRBLUP), “BGLR” for various Bayesian models (e.g. Bayes A, Bayes B, Bayes C and etc.), and “spls” for sparse partial least squares regression model (SPLS), as well as many ML-based methods (Endelman, 2011; Colombani et al., 2012; Blondel et al., 2015; Yan et al., 2021). Although these packages and tools have been used for GS breeding in plants, an integrated environment containing the publically available GS tools may greatly ease the process of users to conduct a sufficient comparative evaluation of GS models (Xu et al., 2021). Additionally, most current tools do not support streamlined pipelines of data preprocessing, model selection and performance evaluation. Data preprocessing is a tedious and complicated process, including data format conversion, data quality control, as well as partition of training and testing samples. Performance evaluation requires complicated fine-tuning of model parameters and objective evaluation with an array of different metrics, such as Pearson correlation coefficient (*r*), Mean square error (MSE). Through these two critical steps, the best models with optimized parameters can then be selected to use for GS-assisted breeding. Because many tools must be configured differently to meet the requirement of specific computing environments, comparative evaluation of different GS models is a complicated process. The Singularity platform, developed as a scientific container for cloud computing, can easily package up tools and software to run in reproducible environments (Kurtzer et al., 2017). Singularity has been utilized to set up cloud computing environments for many bioinformatics tools and pipelines (Iacoangeli et al., 2019; Garofoli et al., 2019; Youens-Clark et al., 2019). Therefore, it provides a promising means to integrate a library of GS models, tools and preconfigured pipelines for streamlined GS analysis. With such an integrated environment, users not only can easily install and run all kinds of GS models, but also can perform streamlined GS analyses without needing advanced programming skills.

In this study, we present the G2P container, developed based on the Singularity platform to serve as a reproducible environment allowing users to perform streamlined GS analysis, including data preprocessing, model construction, and model evaluation. It also allows automatic integration of prediction results to simplify the GS analysis. The library contains up to 16 state-of-the-art GS models and 13 evaluation metrics, which are integrated into a uniform framework to flexibly and conveniently call GS models for comparative evaluation. Moreover, the container-based characteristics of G2P will greatly ease the way for a bioinformatics personnel or population geneticist to perform GS analysis, especially for the situation involving comparative evaluation of multiple GS methods. G2P is not only available as a container, but also can be installed and used as a regular R package. Here, we demonstrate the features and utility of G2P through analysis of a large maize population (CUBIC) comprising 1,404 genotyped inbred lines with three phenotypes (Liu et al., 2020; Luo et al., 2020).

## Methods

### Overall structure of the G2P container

**Figure 1:**
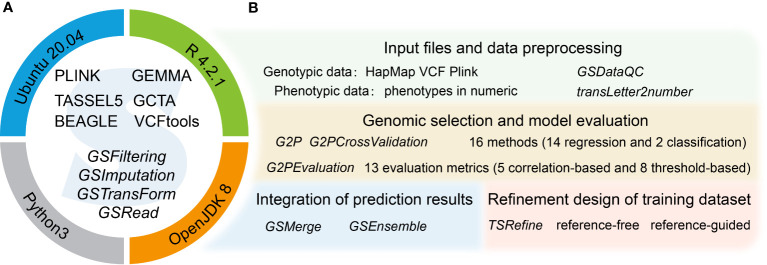
Overview of the G2P singularity container. **(A)** Systems environment setup of the G2P container. The upper semicircle contains several stand-alone software programs (capital letters) for GS related analysis and offering a running environment for the G2P functions (italics) in the lower semicircle. The operating system and computing environment for the G2P container are shown on the circle. **(B)** The four main functional modules of the G2P package, with the main functions of each module highlighted in italics.

The G2P container has two main components. The first comprises stand-alone software and dependent libraries, offering the environment for running G2P (**Figure 1A**). The second component comprises the R package of G2P, containing the main modules required for GS analysis (**Figure 1B**). This consists of four main analytical modules that function as a streamlined pipeline, for preprocessing of genotype and phenotype data (**Supplementary materials**), evaluation of the 16 models in the library, integration of prediction results from multiple models, and refinement of training datasets.

### Parallel evaluation of GS models

The G2P library currently contains 16 models that have been previously used in plant breeding: 14 regression-based models and two classification-based models (**Table 1**). Streamlined pipelines for running these models are integrated into the “G2P” module, which is the core function of the G2P container. A user may specify the models from the library, and the selected models can be simultaneously constructed by setting the parameter “modelMethods” of the “G2P” module. To evaluate the prediction precision of the selected models, 13 evaluation metrics can be called by the function of “G2PEvaluation” (**Table 2**). The 13 evaluation metrics can be classified into two categories: there are five metrics for correlation-based evaluation and eight metrics for threshold-based evaluation. The first five metrics measure the global similarity between predicted and observed phenotypic values with a variety of algorithms to compute correlation, and the remaining eight assess the model precision by examining the proportion of predicted top samples among the actual top samples with user-specified top 10%, 15%, 20%, or 30% of expected phenotypic values (Li et al., 2023).

**Table 1:** Characteristics of the 16 GS models integrated in G2P.

Model	Feature	Package	Refs
GS models based on regression			
Bayes A	Scaled *t*-distribution for marker effects, inverse chi-square on marker variance	BGLR	(de los Campos and Pérez, 2015)
Bayes B	Similar to Bayes A; utilizes both shrinkage and variable selection method		
Bayes C	Characterized by a Gaussian distribution		
Bayesian LASSO (BL)	Marker variances resulting in a double exponential (DE) distribution		
Bayesian ridge regression (BRR)	Induces homogeneous shrinkage of all marker effects towards zero; marker effect yields a Gaussian distribution		
Reproducing kernel Hilbert space (RKHS)	Effective for detecting nonadditive gene effects		
Ridge regression (RR)	Normal distribution for marker effects and nonzero	rrBLUP	(Endelman, 2011)
Ridge regression best linear unbiased prediction (RRBLUP)	Assumes all markers have equal variances with small and nonzero effect		
big ridge regression (bigRR)	An extension and optimization of ridge regression	hglm	(Rönnegård et al., 2010; Shen et al., 2013)
Least absolute shrinkage and selection operator (LASSO)	Combine both shrinkage and variable selection method	glmnet	(Friedman et al., 2010; Ogutu et al., 2012)
Sparse partial least squares (SPLS)	Combining variable selection and modeling in a one-step procedure	spls	(Chung et al., 2012; Colombani et al., 2012)
Support vector regression (SVR)	Finds an appropriate line (or hyperplane in higher dimensions) to fit the data	e1071	(Cortes and Vapnik, 1995; Meyer et al., 2014)
Random forest regression (RFR)	Uses the regression model rooted in bootstrapping sample observations	randomForest	(Breiman, 2001; Liaw and Wiener, 2002)
Bayesian regularization neural networks (BRNN)	Connects two hidden layers of opposite directions to the same output	brnn	(Rodriguez and Flores-Mara, 2019)
GS models based on classification			
Support vector classification (SVC)	Finds an optimal boundary between the possible outputs	e1071	(Cortes and Vapnik, 1995; Meyer et al., 2014)
Random forest classification (RFC)	Uses bagging and feature randomness, consisting of many decision trees	randomForest	(Breiman, 2001; Liaw and Wiener, 2002)

**Table 2:** Descriptions of the 13 evaluation metrics integrated in G2P.

Evaluation Metrics	Description	Range
(I) Correlation-based global metrics		
Pearson correlation coefficient (PCC, *r*, *R*)	Strength and direction of the linear relationship between *X^a^* and *Y^b^*	[-1, 1]
Kendall rank correlation coefficient (KCC, tau, τ)	Ordinal association between *X* and *Y*	[-1, 1]
Spearman rank correlation coefficient (SCC, rho, ρ)	Indicator of how well the relationship between *X* and *Y* can be described using a monotonic function	[-1, 1]
Coefficient of determination, *R* squared (*R* ^2^, *r* ^2^)	Evaluating the goodness of fit of *X* and *Y* of linear regression	[-1, 1]
Mean squared error (MSE)	Average squared difference between *X* and *Y*	[0, +∞)
(II) Threshold-based metrics		
Normalized discounted cumulative gain (NDCG)	Evaluating the prediction ability for selecting individuals that have top-ranked phenotypic values	[0, 1]
Mean normalized discounted cumulative gain (mean NDCG)	Mean of NDCG scores from *k* = 1 to *k = K^c^*	[0, 1]
Relative efficiency (RE)	Expected gains when top *k* individuals are selected	[-1, 1]
Accuracy	Proportion of correctly classified individuals among *K*, for evaluating classification performance	[0, 1]
F-score	Balance between precision and recall	[0, 1]
Area under the receiver operating characteristic curve (AUC)	Area under the receiver operating characteristic curve, for evaluating classification performance	[0, 1]
Area under the precision-recall curve (AUCpr)	Area under the precision-recall curve, for evaluating classification performance	[0, 1]
Cohen’s kappa coefficient (Kappa)	Evaluating the agreement between *X* and *Y*	[-1, 1]

a Observed phenotype. ^b^Predicted phenotype. ^c^Total number of predicted individuals.

Comparative evaluation of multiple GS methods using a cross-validation (CV) scheme can be executed by the module “G2PcrossValidation,” and this may also facilitate the selection of the optimal parameters to achieve the best model performance. Because multiple GS models and numerous folds of CV are simultaneously evaluated, “G2PCrossValidation” is also empowered with parallel computation on high-performance computing clusters to accelerate the computing efficiency. It is also worth noting that both the “G2P” and “G2PCrossValidation” modules allow users to construct more complex models by including other information besides genotypes. After a comprehensive evaluation of the GS models in the library of G2P, the final result of the evaluated GS models is presented so that users may select the most appropriate model or models for selecting breeding materials.

### Integration of multi-model prediction results

Because no single model works best for all species and traits, aggregating the results from multiple GS models will further improve the precision of phenotype prediction. G2P offers two strategies, GSMerge and GSEnsemble, to integrate multi-model results, using the functions “GSMerge” and “GSEnsemble,” respectively. The development of the GSMerge algorithm adopted the idea of maximal accuracy–maximal difference (MAMD) based on the evaluation results of different models. First, after phenotype prediction, a matrix of predicted phenotypes multiplying GS models is constructed. Second, the models are ranked based on their overall prediction precision evaluated by one metric (e.g., Pearson correlation coefficient) to generate a ranking, *r1*, and the top model is selected. Third, predicted phenotypic values of the top model are extracted, and then the correlations of its values with the values predicted by the other models are calculated. This generates a new ranking, *r2*. Fourth, the difference between the rankings *r1* and *r2* is calculated, and then a second model is selected that shows not only the best prediction precision but also the maximal difference from the top model. Finally, a new value is computed as the integrated prediction results from averaging the predicted values of the two selected models. In addition to this automatic integration process, the “GSMerge” function can also execute self-defined integration with provided weights from “GSEnsemble” described below or own experience.

GSEnsemble adopts another strategy, which is to perform multiple rounds of iteration and repetition to integrate multi-model results. GSEnsemble takes the same matrix of prediction results from multi-model analysis as the initial input, and then a list of initial weights computed based on the evaluation of model precision is automatically generated. Subsequently, the self-defined GSMerge integration procedure is applied to the two models that are to be integrated. The difference is that GSEnsemble integrates results by considering the more possible weights of the two models instead of simply averaging the results from the two models. Then, after evaluation and selection, the new result from the first two models is merged with the result from the third model and the corresponding weights are recorded. These integration steps are repeated until the results from all of the models are applied to the iteration and a list of optimal weights is finally generated. It is worth noting that, rather than using one weight combination, GSEnsemble allows users to define the time of repetitions (i.e. the order in which the various models are integrated) to shuffle the input of the result matrix and obtain a weight matrix. Therefore, every set of weight combinations can be used to compute a grand phenotypic value using weighted prediction results of all the GS models in the library. Simply speaking, the fundamental difference between GSMerge and GSEnsemble is that GSEnsemble first generates a list of the optimal combinations of models and weights by a series of iterations and then computes the grand value based on the weighted results of all of the models. However, GSEnsemble processing is slower than GSMerge processing for large training populations.

### Refinement design of training dataset

Compilation of an appropriate training set is extremely important for model precision and robustness, as the training set should offer sufficient genetic diversity and coverage in sample prediction. Otherwise, it may cause problematic either underfitting or overfitting issues. With the accumulation of more and more samples that are both genotyped and phenotyped, the training dataset will be refined by both selecting representative samples and removing redundant samples. G2P offers the module “TSRefine” to allow both reference-free and reference-guided algorithms to refine the training dataset. The reference-free algorithm uses the concept of the minimum moment aberration (MMA) algorithm to select a subset of candidates with the maximum discrepancy of genotypes. MMA measures the discrepancy between two individuals in their marker genotypes by averaging all pairwise similarities (Jin et al., 2004; Sen et al., 2007). In this manner, the refined training dataset may present more representative genetic diversity than the original training set. A reference-free algorithm is preferable when genotypes are difficult to obtain. In contrast, the reference-guided algorithm utilizes the testing dataset as a reference. Through the construction of two matrices of genomic relationship, one within the training set and another between individuals in the testing set with the training set. Also, three alternative criteria, “PEVmean,” “CDmean,” and “Sim,” are used as measurable criteria to seek the optimal solution until the iteration becomes stable (Akdemir et al., 2015).

## Results

### Demo datasets for developing the G2P container

To illustrate the utility and performance of the G2P container, we used a previously published dataset of maize inbred lines, the Complete-diallel plus unbalanced breeding-derived inter-cross (CUBIC) population, for testing (Liu et al., 2020). The CUBIC population contains 1,404 recombinant lines produced from 24 elite inbred lines that have been widely used for maize breeding in China (Liu et al., 2020; Luo et al., 2020). The raw genotypic data, in the binary PLINK format, of the 1,404 lines contains 14 million single-nucleotide polymorphisms (SNPs) called from the whole-genome resequencing of the CUBIC population. The phenotypes used as the target traits for GS prediction include days to tassel (DTT), plant height (PH), and ear weight (EW), representing the three critical developmental stages of maize development. We focused on EW because it is an important trait for evaluating maize yield and one of the most complex and difficult traits to predict due to its low heritability. Members of the CUBIC population were planted in five ecological regions to collect raw phenotypic data, which were further analyzed by the best linear unbiased predictor (BLUP) model to remove variation caused by environmental effects. The original datasets and the details of genotype and phenotype collection and processing can be found at the website http://cubicmaize.hzau.edu.cn and in the original research articles (Liu et al., 2020; Luo et al., 2020).

### Parallel evaluation of the 16 GS models using 13 evaluation metrics

**Figure 2:**
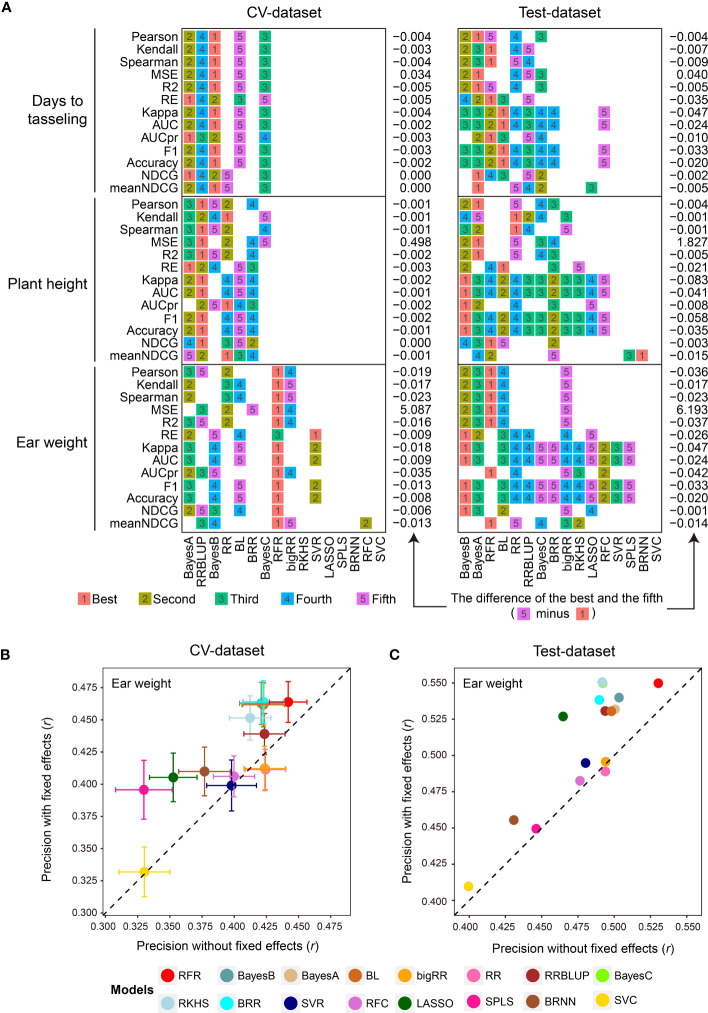
Parallel evaluation of the GS models. **(A)** The ranks of prediction precisions for days to tasseling (DTT), plant height (PH), and ear weight (EW) of 1,404 maize inbred lines on the CV-dataset and Test-dataset by the 16 GS models with 13 evaluation metrics. **(B, C)** Prediction precision for the EW trait using the 16 GS models. DTT and PH were considered as fixed effects, and the 9,286 SNPs were considered as random effects, on the CV-dataset and Test-dataset, respectively. The precision was evaluated based on the Pearson correlation coefficient (*r*).

Subsequently, the 1,000 training samples in the CV-dataset were used for comparison with the 16 GS models in the library using the 13 evaluation metrics by 5-fold CVs with 50 replications (**Supplementary Table 1**). As we expected, the 16 GS models showed greatly varied prediction precisions for the three traits. The RFR, Bayes B, and RRBLUP methods achieved the best performance using the most evaluation metrics when predicting EW, DTT, and PH phenotypes (**Figure 2A**). For both datasets, the 16 GS models yielded varying precision, with Pearson correlation coefficient (*r*) values ranging from 0.323 to 0.443 when predicting EW (**Figures 2B, C**). Among them, the RFR model ranked as the best method, as it achieved the best performance using all five of the correlation-based methods. Bayes B was the best choice if using metrics to predict the top 30% of samples (**Figure 2A**). For the other two traits, Bayes A and BL were the top two models for predicting DTT, while Bayes A and Bayes B were the top two for predicting PH (**Supplementary Table 2**). This result is consistent with the previous reports that no single method is best for all species and traits, such that careful model selection is critical for effective GS-assisted breeding (Ornella et al., 2014; Yan et al., 2021; Robert et al., 2022). It also highlights the need to integrate the results from multiple evaluation metrics to achieve unbiased selection of the optimal GS models for a specific target trait in a designated species.

Perhaps one of the most important factors affecting model performance is the sample size of the training population (Wray et al., 2013; Jiang et al., 2020). To address this, G2P offers an optional function of inferring the optimal population size for model training by using different gradients of the numbers of training samples set by the users. In our demo analysis, we gradually expanded the training set from 200 to 1,000 samples, and then visualized the prediction precision of the 16 models in terms of the Pearson correlation coefficient (*r*) of the three traits with boxplots and line charts (**Supplementary Figure 1**). Overall, all three traits showed a similar trend whereby the prediction precision of the 16 models gradually improved with the expansion of the training set—for DTT, from 0.429 to 0.603; for PH, from 0.453 to 0.619; and for EW, from 0.280 to 0.478. Furthermore, because considering additional fixed effects in a GS model may also improve prediction precision (Sarinelli et al., 2019), G2P allows users to add customized fixed effects to the GS model. In the demo analysis, we utilized DTT and PH as fixed effects in addition to the 9,286 SNPs and then performed 5-fold CV with 50 replicates on both the CV-dataset and Test-dataset to evaluate the models (**Supplementary Table 3**). In comparison with the results generated from the model only using SNPs as random effects, the prediction precisions of 14 of the 16 models (the exceptions being RR and bigRR) were elevated. Among them, SPLS and LASSO showed the greatest improvement, increasing by 0.066 (*r*: 0.330 to 0.396) and 0.062 (*r*: 0.465 to 0.527) accuracy, respectively (**Figures 2B, C**). It is also worth mentioning that G2P allows batch submission of the 16 GS models and the 13 evaluation metrics for parallel evaluation if a cluster of high-performance computers is available. This functionality offers the greatest convenience to the users for comparative evaluation of GS models to assist selection of the optimal GS models.

### A benchmark test of analytical efficiency of the 16 models

In addition to prediction precision, analytical efficiency is another important factor to consider, especially when the sizes of samples and features are large. G2P records the CPU run time and memory usage when performing model evaluation. In this study, we used the benchmark test to evaluate the analytical efficiency of the 16 GS models under the G2P Singularity environment on a Windows desktop with a configuration of 4-core CPU i5 6600K and 32 GB memory. The benchmark test was performed on the Test-dataset containing the 1,000 samples to record CPU run time and memory usage, and this process was repeated ten times to calculate the average efficiency.

**Figure 3:**
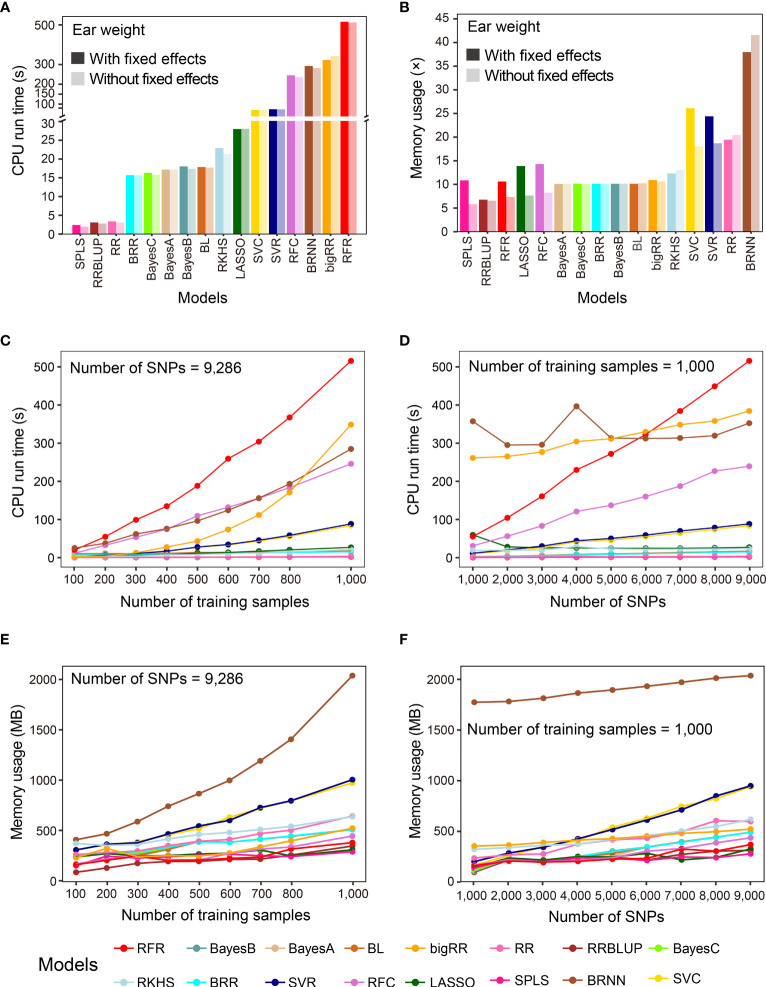
Benchmark test of the analytical efficiency of the 16 GS models. **(A, B)** Comparison of results after running the 16 GS models for training and prediction on the CV-dataset in terms of CPU run time **(A)** and memory usage **(B)**. CPU run time is indicated in seconds (s) and memory usage in multiple (×) of running memory over initial data memory. **(C, D)** Correlation of CPU run time with the number of training samples **(C)** and the number of SNPs **(D)**. **(E, F)** Correlation of memory usage in megabytes (MB) with the number of training samples **(E)** and the number of SNPs **(F)**.

The averaged run times of the 16 models varied greatly (**Figure 3A**). The top three methods were SPLS, RRBLUP and RR, with run times of less than 3.5 seconds. BRR, Bayes A, B and C, and BL needed less than 20 seconds, and the other 8 methods all took more than 100 seconds. It is worth mentioning that, although RFR achieved the best precision when predicting EW, it took the longest, at 529.9 seconds. Memory usage was evaluated using a method called multiple (×) of initial data memory usage, which was 50.54 Mb when the entire dataset was loaded into memory. If no fixed effect was added, SPLS, RRBLUP, RFR, LASSO, and RFC showed the lowest memory usage, below 10×, and the top four memory-usage models were BRNN, RR, RFC and RFR, using 41.56×, 20.44×, 18.05×, and 18.70× memory, respectively (**Figure 3B**). When fixed effects were added to the models, memory usage increased in all cases, but to different degrees for most of the models. Finally, we examined how the sample size and marker number influence CPU run time and memory usage of the 16 GS models, finding that both sample size and marker number were positively correlated with the computing resource for most models (**Figures 3C-F**). Especially for RFR and RRBLUP, CPU run time and memory usage of RFR were both linearly influenced by marker number and RFR was linearly influenced by sample size.

### Integration of multi-model prediction results

**Figure 4:**
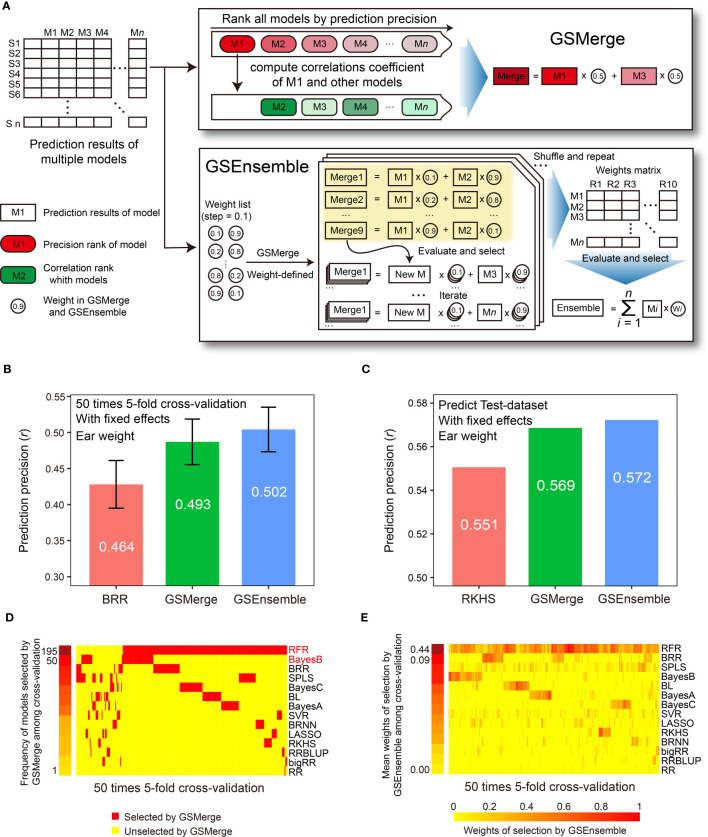
Improvement of prediction precision for the EW (ear weight) trait through integration of multi-model results. **(A)** Schematic illustration of the algorithms for the two result integration functions. GSMerge selects models according to the difference of two ranks (ranks of prediction precision and ranks of correlation coefficients between top1 model and others). Integration results of GSMerge is the average of prediction results of two selected models. The deeper the color, the higher the correlation. By generating a weight list and executing GSMerge, GSEnsemble selects models and gets weights of models after multiple iterations and repetitions. Integration results of GSEnsemble is the weighted average of all models. **(B)** Prediction precisions by GSMerge- and GSEnsemble-based integration are better than those for the single model, BRR, with fixed effects considered on the CV-dataset. The prediction precision was evaluated based on the Pearson correlation coefficient (*r*). **(C)** After integrated combination and weights from the CV-dataset are utilized on predicting Test-dataset, prediction precisions for GSMerge- and GSEnsemble-based integration are improved relative to those predicted by the best single model, RKHS, with fixed effect considered. **(D)** The statistics for the frequency of models selected by the GSMerge strategy using 50 times 5-fold cross validation on the CV-dataset. **(E)** The statistics of the weights of the models used for GSEnsemble on the CV-dataset.

Overall, the prediction precision varied from model to model, but certain models may generate similar predicted results. This is reflected in the pairwise correlations (ranging from 0.54 to 1.00) computed between the predicted phenotypic values by any pair of models. This indicates that complementary relationships may exist between certain GS models, which we may take advantage of to integrate multi-model prediction results. G2P offers two strategies for integrating predicted results from top-precision models, the GSMerge and GSEnsemble algorithms (**Figure 4A**). GSMerge automatically selects two top models with relatively high precision and considerable difference in output. It then directly averages the two sets of prediction results to generate a single integrated result. In contrast, GSEnsemble considers the results from all available models, after a search of a set of optimal weights by iteration and repetition, to generate a grand value by summarizing weighted values of all the models (See Methods).

Within the CV-dataset, we tested the effectiveness of integration of multi-model predictions by the two algorithms for each fold of CV. As we anticipated, when considering fixed effects, both GSMerge and GSEnsemble strategies improved the precision of EW prediction compared to the single BRR model that was the best predictor, increasing the *r* value from 0.464 to 0.493 and 0.502, respectively (**Figure 4B**). Consistent results were also observed when fixed effects were not considered. The precisions of GSMerge and GSEnsemble also increased, from 0.443 (RFR model) to 0.445 and 0.465, respectively (**Supplementary Figure 2A**). Furthermore, the frequency of models selected by GSMerge among the 50 times 5-fold (a total of 250 repetitions) were summarized to help direct model selection for integration of the prediction results for the Test-dataset (**Figure 4D**). For GSEnsemble, the mean weights of all 250 repetitions were calculated to obtain a set of weights of the models to further utilize GSEnsemble for integrating the results from the Test-dataset (**Figure 4E**). The same test of GSEnsemble-based integration was performed without fixed effects, and the outcome was consistent with the outcome when considering fixed effects (**Supplementary Figure 2**). Therefore, the summarized results not only give the users a reference for integration of multi-model prediction, but also assist users in understanding which model contributes the most weight under results integration.

To verify the effectiveness of prediction result integration, we used the two most commonly used models (RFR and BayesB) for GSMerge on the CV-dataset to perform GSMerge on the Test-dataset as well as mean weights list for GSEnsemble on the CV-dataset to perform GSEnsemble on the Test-dataset. As we anticipated, both the GSMerge and GSEnsemble algorithms improved the prediction precision on the EW traits compared to the single models RFR and RKHS with and without fixed effects considered, respectively (**Figure 4C**; **Supplementary Figure 2B**). It’s also worth of noting that improvement of prediction precision by GSEnsemble was better than that for GSMerge, regardless of whether fixed effects were considered. These results indicate that multi-model integration is a practical, effective strategy to achieve better prediction of desired phenotypes.

### Application of G2P container on real-world breeding data

We applied the G2P container on two sets of real-world breeding data provided by a collaborating seed company. The first dataset (Dataset 1) contained 7, 046 F_1_ hybrids generated by crossing 3, 523 inbred lines with 2 tester lines, which were planted in the ecological zone of Northeast China in 2021 and 2022 to collect phenotypes. The second dataset (Dataset 2) contained 6,777 F_1_ hybrids generated by crossing 2, 259 inbred lines with 3 tester lines, which were planted in Central China in 2021 and 2022 to collect phenotypes. The total 5, 782 inbred lines and the 5 tester lines were genotyped using the genotyping by targeted sequencing (GBTS) platform containing 44, 229 SNPs. We first analyzed the genotypic data of the 5, 787 samples, and simulated the heterogzygous genotypes of the total 13, 841 F_1_ hybrids, utilizing the preprocessing pipeline in the G2P container described in **Supplementary Materials**. The entire streamlined procedure of genotypic data analysis took about ten minutes on a desktop server (Xeon-E5 with 4-core and 64 GB memory). The raw phenotypic data of grain yield (GY) per unit collected in the two years and six locations planted in each of the ecological zones were processed by the BLUP algorithm.

After the genotypic and phenotypic data were processed, we then performed the GS analysis module with the 16 GS models and 13 evaluation metrics. We randomly selected 2, 046 and 1, 777 F1 hyrbids from the Dataset 1 and 2 as external validation set, respectively. Then, we utilized the 5, 000 samples in each of the datasets to train and evaluate models, followed by predicting the GY per unit of the samples in the two external validation set. At last, the predicted GY phenotypes of the validating samples were generated by the GSEnsemble and GSMerge algorithms to integrate multi-model prediction results. The GS analysis procedure took about tree minutes on the same server to generate the model evaluation result (**Supplementary Table 4**). The evaluation results showed that RRBLUP method exhibited relatively stable and precise prediction results compared to other methods in the Dataset 1, while the RKHS method outperformed other GS models. We then utilized the two algorithms, namely GSMerge and GSEnsemble developed in G2P, to integrate multi-model prediction results. For the Dataset 1, the prediction precision were improved from 0.500 (RKHS model) to 0.519 (GSEnsemble) evaluated by Pearson correlation coefficient (**Supplementary Figure 3A**). For the Dataset 2, the prediction precision were improved from 0.503 (RRBLUP model) to 0.530 (GSEnsemble) evaluated by Pearson correlation (**Supplementary Figure 3B**). This result indicates that integration of multi-model prediction result may greatly improved prediction precision.

### Refinement design of training dataset

**Figure 5:**
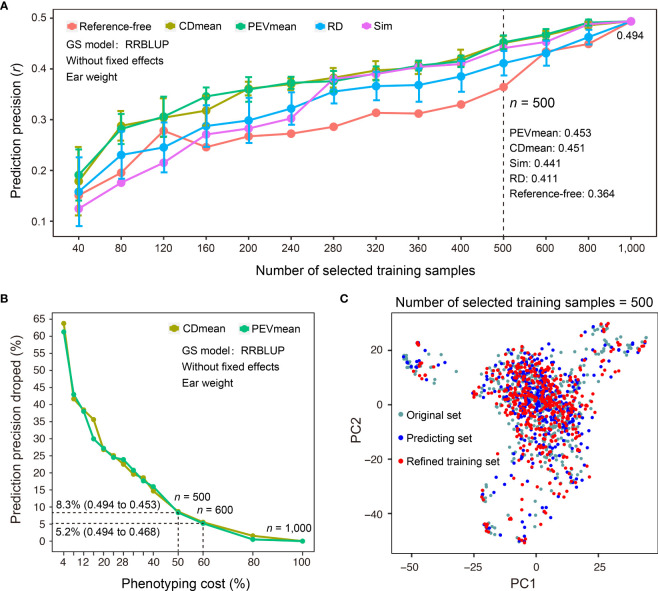
Comparison of different methods for refinement design of the training dataset. **(A)** Comparison of prediction precisions of the Test-dataset on EW (ear weight) between the training sets compiled by TSRefine selection (Ref-free for reference-free method, CDmean, PEVmean and Sim for reference-guided method) and random selection (RD) from the CV-dataset. RRBLUP was the GS model used to perform prediction from refined training set to Test-dataset and prediction precision evaluated based on the Pearson correlation coefficient (*r*). **(B)** Percentage decrease in prediction precision over the highest precision using different numbers of samples compiled by CDmean and PEVmean. Phenotyping cost represents the percentage of TSRefine selection in the entire CV-dataset (*n* = 1,000, where *n* is the number of selected samples). **(C)** Comparison of the genetic distributions of the original, prediction, and refined training samples by PCA.

In addition to sample size, selecting a representative set of training samples while achieving the maximal coverage of genetic diversity in predicting samples for which phenotypes are not measured is a critical step in GS-assisted breeding. Three main advantages may benefit from refinement design of the training dataset based on the genotypes of samples: first, it can decrease the cost of phenotyping when a subset of training samples is selected; second, it may avoid model overfitting if the training samples do not properly encompass the genetic diversity of predicting samples; third, training samples may continuously grow during the actual breeding practice, and it becomes necessary to continually reevaluate the genetic structure of newly phenotyped and genotyped samples. To assist the refinement design of a training dataset, G2P offers the TSRefine module, which facilitates selection of a subset of samples from the total training set using reference-free and reference-guided algorithms (See Methods). To test the effectiveness of the TSRefine function, we compared the prediction precisions using a variety of training set sizes by random selection (RD) and TSRefine selection of 40 to 1,000 samples. The comparison showed that the PEVmean and CDmean of RRBLUP have an overwhelming advantage over those for random selection (**Figure 5A**). On average, prediction precisions using the training set by TSRefine selection were 0.051 to 0.056 higher than those obtained using random selection evaluated by the Pearson correlation coefficient (*r*). When TSRefine selected 500 representative training samples using the reference-guided model (PEVmean), the prediction precision on the Test-dataset dropped only 8.3%, from 0.494 to 0.453, compared to the precision using the entire set of the 1,000 samples (**Figure 5B**). This indicates that phenotyping cost may be reduced to half with only an 8.3% sacrifice in precision.

Additionally, TSRefine can illustrate the distribution of the original, predicting, and refined training sets using a scatter plot generated by PCA. With this, users can better understand the genetic relationship of the above three sets in the background of the whole population structure (**Figure 5C**). If no genotypes of predicting samples are available, an alternative is to utilize a reference-free mode of refinement design of training dataset. When 600 representative samples were selected to compile an optimized training set, the prediction precision on the Test-dataset dropped only 5.2%, from 0.494 to 0.468 (PEVmean; **Figure 5B**). Notably, however, this strategy can be applied only when the training and prediction samples are largely similar in terms of genetic backgrounds. Otherwise, it may cause either model overfitting or underfitting.

## Discussion

GS has become a novel breeding strategy that is considered to be an important driving force for the new era of crop breeding (Jiang et al., 2020). Many statistical and informatics methods have been utilized to develop GS models to facilitate prediction of phenotypes from genotypes. The most popular methods include best linear unbiased predictor (BLUP)-based, Bayesian-based and machine learning-based methods. However, because of the sophisticated genetic complexity of crop species, no single method will work best for all species and traits. Model selection to determine the most appropriate model based on the particular traits and species therefore is a crucial step in GS. To solve this challenge, we utilize the Singularity platform to develop the G2P container, which packages up a library of 16 state-of-the-art GS models and 13 evaluation metrics. The main function of G2P is to perform batch evaluation of multiple GS models with uniformed analytical framework assisted by parallel computing. This utility offers great convenience for users by enabling them to perform comprehensive and unbiased model evaluation without having programming experience.

Another important feature of G2P is its use of two strategies to integrate multi-model prediction results in an automatic fashion. Our analysis showed that result integration may effectively improve prediction precision. G2P also offers the capability of conducting refinement design of training datasets, allowing selection of a subset of optimized training samples based on genetic diversity analysis of training and predicting populations. This function is quite useful in practice, as it can not only greatly reduce the cost of phenotyping when constructing training population, but also help design a GS project amid the continuous growth of breeding data with available genotypes and phenotypes. In addition to its main function of GS analysis, G2P also provides a series of bioinformatics tools for data preprocessing including data filtration, file format conversion, representative SNP selection, genotype and phenotype imputation, and data quality control. These tools greatly simplify the operation for users without much programming skill.

Taking the advantages of Singularity platform, the G2P container is flexible and easy to install and to upgrade, but limitations still have to be noticed. First of all, the purpose of developing G2P container is provide a convinent environment which may simplify the procedure of installing GS tools and performing GS analysis, so that starter users without sufficient experinces in GS analysis and programming skills may use it. Thus, only a limited number of GS tools are packaged with the Singularity platform, which are easy to be developed as standard, automated pipeline. Many of machine learning or deep learning (DL)-based GS tools were not integrated into G2P container, as these methods requires sophisticated parameter tuning or GPU computing. If an experienced user wants to perform ML or DL-based GS analysis, standlone tools are highly recommended. Secondly, because one of the novel features of G2P container is to integrate prediction results from the 16 GS models, computing efficiency is also an important factor that have to be considered. Thus, preprocessing of training dataset is highly recommended to compile a marker panel better within 10K SNPs, and the training population better contains less than 10K samples. If a user wants to perform GS analysis on much larger sample set with a marker panel containing over tens of thousands of SNPs, it’s better to use standalone version of GS tools. Morever, the current analytical pipelines in G2P only consider additive effects when training a BLUP-based GS model and epistatic interactions between SNPs were not considered, as modulating of epistatsis may expotentially increase computational complexity causing unexpected error during model training. At last, the current version of G2P container only integrate regression-based methods that are mostly used for predicting quantatitve traits of common crop species, without considering special scenarios or traits subjected to GS analysis. To cope with the above-mentioned limitations, we plan to continuously upgrade the G2P container in the near future, with more GS algorithms, more analytical functions, and more user-friendly data input such as relationship matrix or kinship matrix. In the future, G2P will be developed as a workshop for crop breeders who wish to conduct cloud-based precision-designed breeding.

## Data availability statement

Publicly available datasets were analyzed in this study. The raw datasets can be found at http://cubicmaize.hzau.edu.cn/ and the filtered data used in this study can be found at https://g2p-env.github.io/ or built-in dataset for example.

## Author contributions

QC and SQJ conceived and supervised the project. QC, SQJ, XFW and MC wrote the manuscript. QW, ZXQ and QC developed the main G2P packages and performed demo analysis using the maize CUBIC population. SQJ and SJ developed the pipelines for data preprocessing. TL developed the evaluation modules. ZXQ, JY and RF performed the benchmark test of G2P. All authors read and approved the final manuscript.

## Supplementary material

Supplementary Figure 1 Comparison of the prediction precision on the Test-dataset for DTT (days to tasseling), PH (plant height), and EW (ear weight) got the 16 GS models overall **(A)** and separately **(B)** using different numbers of training samples.

Supplementary Figure 2
**(A)** Prediction precisions by GSMerge- and GSEnsemble-based integration are better than those by the single model RFR without fixed effects considered on the CV-dataset. The prediction precision was evaluated based on the Pearson correlation coefficient (*r*). **(B)** By applying integrated combination and weights from the CV-dataset to prediction of the Test-dataset, prediction precisions with GSMerge- and GSEnsemble-based integration are more improved than those with the best single model, RFR, without fixed effects considered. **(C)** The statistics of model selection frequency by the GSMerge strategy through 50 times 5-fold cross validation on the CV-dataset. **(D)** The statistics of the weights of the 14 models used for GSEnsemble strategy on the CV-dataset.

Supplementary Figure 3 The prediction precision was evaluated by Pearson correlation coefficient of two results integration methods on real-world breeding data. **(A)** The ecological zone of Northeast China and **(B)** The ecological zone of Central China
