# Supplementary figures and images for: G2P Provides an Integrative Environment for Multi-model genomic selection analysis to improve genotype-to-phenotype prediction
Source: Front Plant Sci. 2023 Aug 4;14:1207139. doi: 10.3389/fpls.2023.1207139 (PMC10437076; doi:10.3389/fpls.2023.1207139)

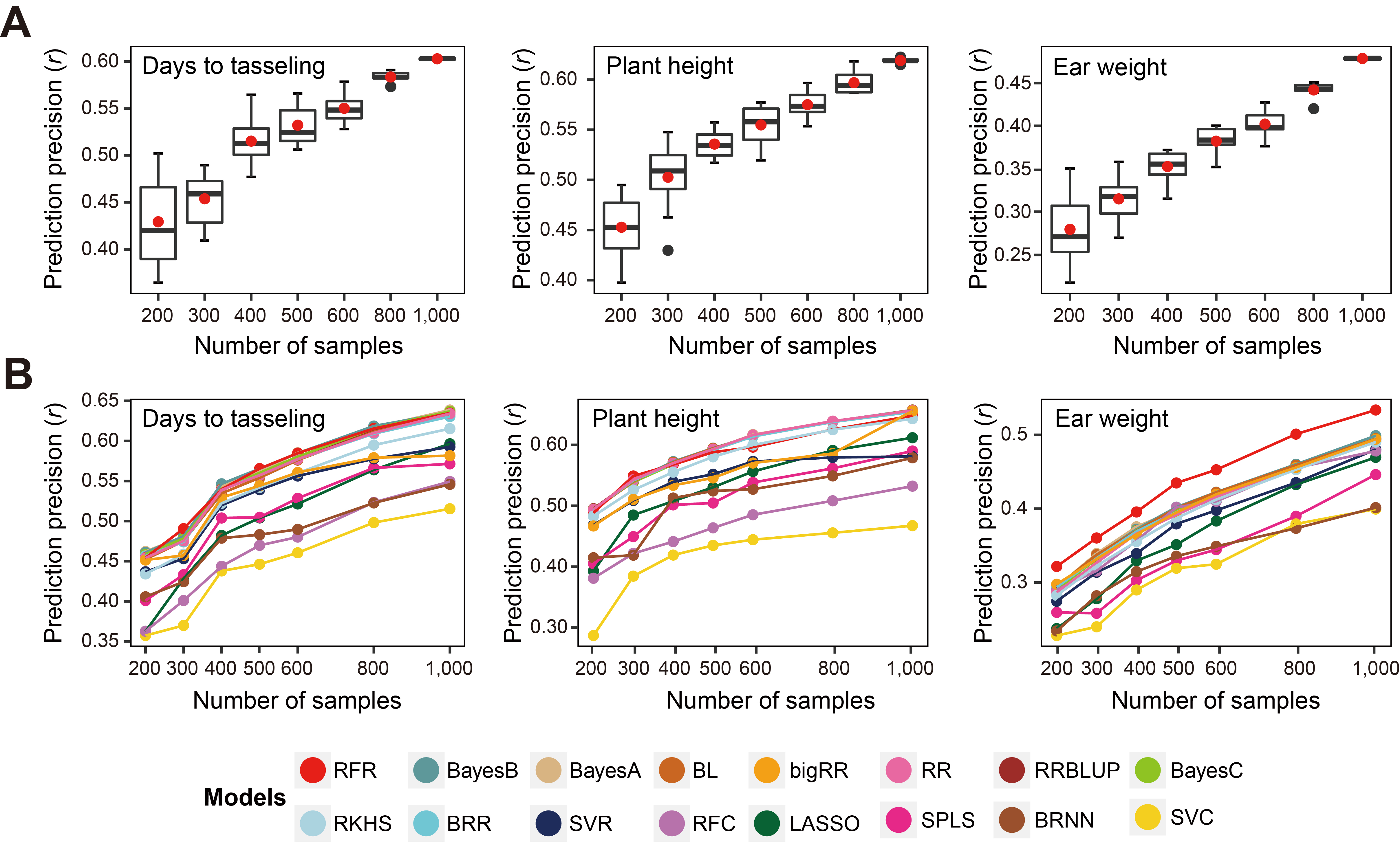

Supplement: Supplementary Figure 1 — Comparison of the prediction precision on the Test-dataset for DTT (days to tasseling), PH (plant height), and EW (ear weight) got the 16 GS models overall (A) and separately (B) using different numbers of training samples. [file Image_1.jpeg]

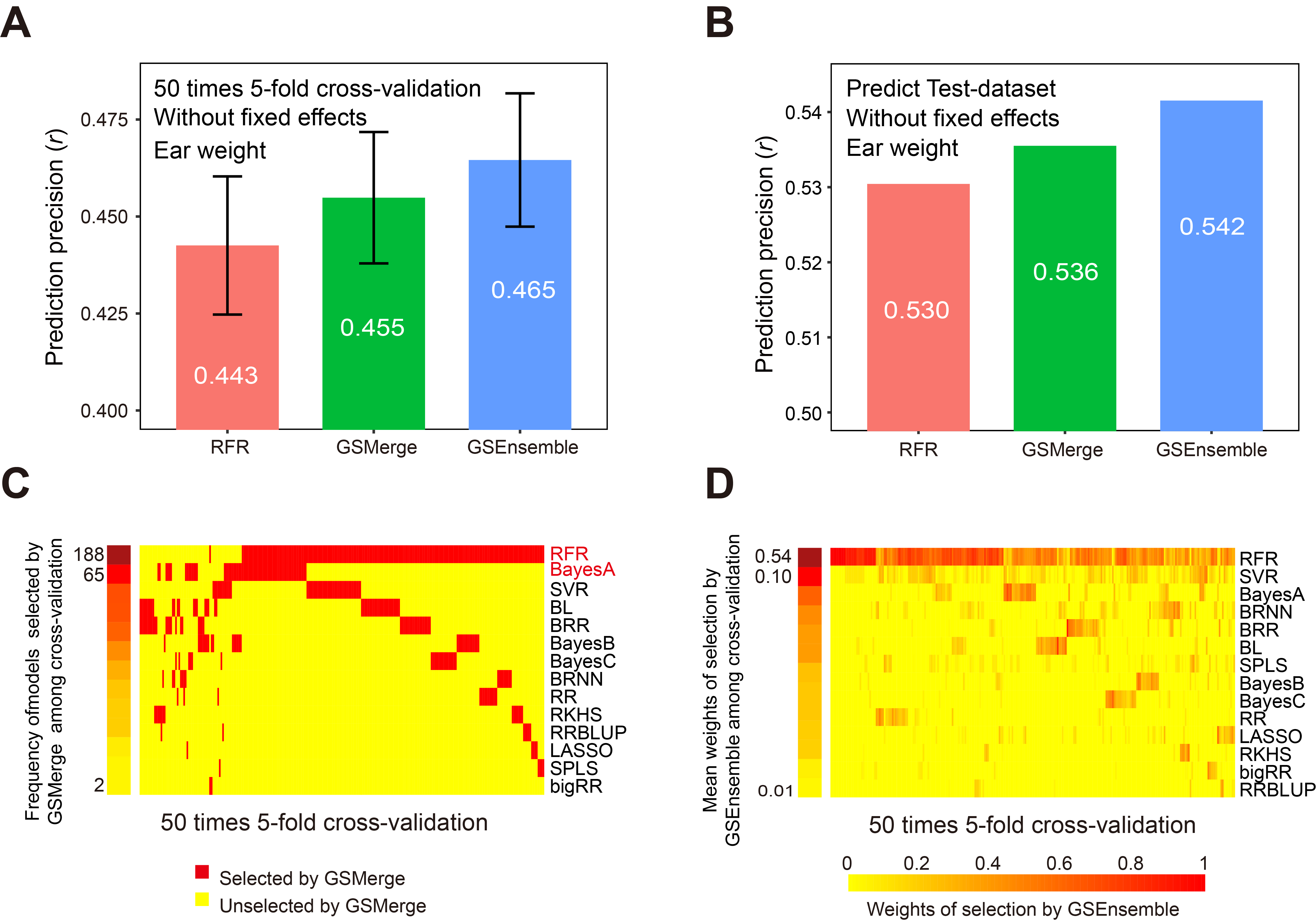

Supplement: Supplementary Figure 2 — (A) Prediction precisions by GSMerge- and GSEnsemble-based integration are better than those by the single model RFR without fixed effects considered on the CV-dataset. The prediction precision was evaluated based on the Pearson correlation coefficient (r). (B) By applying integrated combination and weights from the CV-dataset to prediction of the Test-dataset, prediction precisions with GSMerge- and GSEnsemble-based integration are more improved than those with the best single model, RFR, without fixed effects considered. (C) The statistics of model selection frequency by the GSMerge strategy through 50 times 5-fold cross validation on the CV-dataset. (D) The statistics of the weights of the 14 models used for GSEnsemble strategy on the CV-dataset. [file Image_2.jpeg]

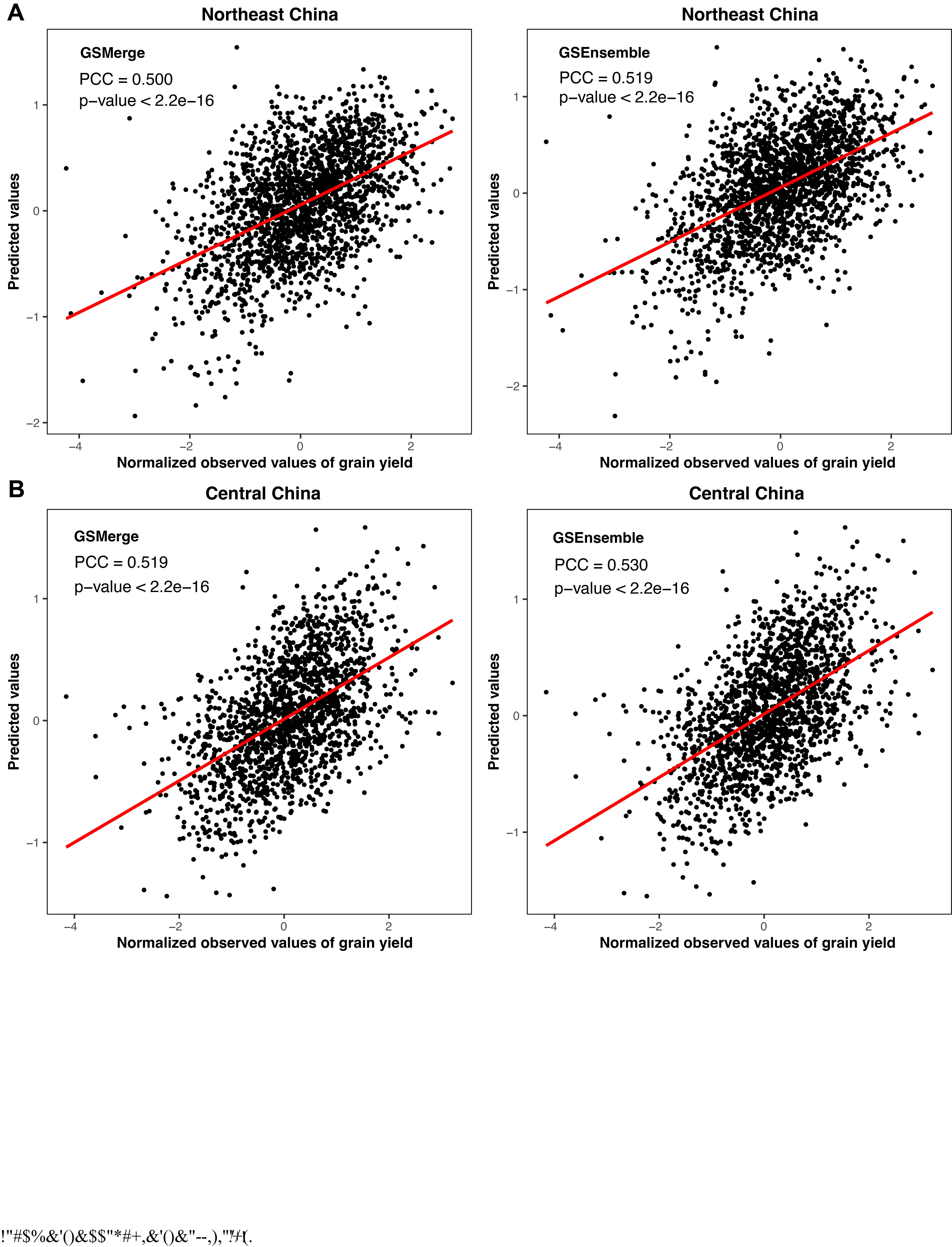

Supplement: Supplementary Figure 3 — The prediction precision was evaluated by Pearson correlation coefficient of two results integration methods on real-world breeding data. (A) The ecological zone of Northeast China and (B) The ecological zone of Central China [file Image_3.jpeg]
