# Supplementary material for: G2P Provides an Integrative Environment for Multi-model genomic selection analysis to improve genotype-to-phenotype prediction
Source: Front Plant Sci. 2023 Aug 4;14:1207139. doi: 10.3389/fpls.2023.1207139 (PMC10437076; doi:10.3389/fpls.2023.1207139)
Supplement: Supplementary file 4 [file DataSheet_1.docx]

**Supplementary Materials**

**G2P Provides an Integrative Environment for Multi-model Genomic Selection Analysis to Improve Genotype-to-phenotype Prediction**

Qian Wang^1,2#^, Shan Jiang^1,2#^, Tong Li^1,2^, Zhixu Qiu^3,4^, Jun Yan^1,2^, Ran Fu^1,2^,Chuang Ma^3,4^, Xiangfeng Wang^1,2^, Shuqin Jiang^1,2*^, Qian Cheng^1,2*^

^1^ Frontiers Science Center for Molecular Design Breeding, China Agricultural University, Beijing 100193, China.

^2^ National Maize Improvement Center of China, College of Agriculture and Biotechnology, China Agricultural University, Beijing 100193, China.

^3^ Key Laboratory of Biology and Genetics Improvement of Maize in Arid Area of Northwest Region, Ministry of Agriculture, Northwest A&F University, Yangling, Shaanxi 712100, China.

^4^ State Key Laboratory of Crop Stress Biology for Arid Areas, Center of Bioinformatics, College of Life Sciences, Northwest A&F University, Shaanxi, Yangling, 712100, China

^#^ These authors contributed equally.

^*^ Correspondence should be addressed to: Shuqin Jiang ([wanshi0066@126.com](mailto:wanshi0066@126.com)) and Qian Cheng ([qchengray@cau.edu.cn](mailto:qchengray@cau.edu.cn)).

**Environment and tools required for installing G2P**

The Linux operation system Ubuntu (version 20.04) is used as the basic system environment for the G2P container, and the R (version 4.2.1) is the computing environment to run the packages. Python3 and OpenJDK are also installed to provide the environment for running stand-alone software, such as PLINK for genetic analyses, GEMMA for genome-wide association studies, TASSEL for trait association analyses, evaluation and linkage analyses, GCTA for genome-wide complex trait analyses, BEAGLE for genotype calling and imputation, and VCFtools for processing variant call format (VCF) files (Bradbury et al., 2007; Browning and Browning, 2007; Purcell et al., 2007; Danecek et al., 2011; Yang et al., 2011; Zhou and Stephens, 2012). The above-mentioned software can be normally used in shell of the container and can also be called by several functions within G2P.

**Preprocessing pipeline of input genotypic data**

The genotypic and phenotypic datasets are the basic input files for G2P, which takes various commonly used formats of genotypic data files, such as PLINK, VCF, and HapMap formats; it first utilizes the module “GSRead” to convert these formats to a numeric matrix for the subsequent analysis. To cope with large volumes of genotypic data (e.g., if millions of SNPs and thousands of samples are included in one file), G2P invokes the package “data.table” to accelerate the reading of the data file into memory from a hard disk. To filter raw SNP dataset, the module “GSFiltering” can be called from the G2P container. This filters the SNPs using several thresholds commonly used in population genetics analysis, such as minor allele frequency (MAF), missing rate, Hardy-Weinberg equilibrium (HWE), and linkage disequilibrium (LD). After the SNP set is filtered and condensed, performing quality control of genotypic and phenotypic data using the module “GSDataQC” is strongly suggested. Besides verifying the integrity of input data files, one important function of “GSDataQC” is to perform imputation to fill in missing values of genotypes. After a new genotypic dataset of a set of greatly condensed high-quality SNPs is generated, “GSTransForm” and “transLetter2number” are run jointly to transform the character-based genotypes (A, C, G, T) in HapMap format to a numeric genotype format (0, 1, 2).

**Preprocessing of demo genotypic data with G2P**

To ensure analytical efficiency and good performance of GS prediction, the raw genotypic data must be preprocessed through multiple steps, including file format conversion, removal of low-quality and excessive SNPs, and imputation to infer the missing genotypes of certain SNPs. Excessive SNPs and low-quality data not only add extra computational burden but also cause biased estimation of marker effects. Especially for certain ML-based models, excessive SNPs as a feature set may cause dimensionality explosion (Yan and Wang, 2022a, 2022b). Thus, a highly condensed SNP set containing approximately 10,000 SNPs is sufficient for GS prediction, considering both analytical efficiency and model precision (Jiang et al., 2020; Yan et al., 2021). G2P includes a series of modules (GSRead, GSFiltering, GSTransForm, and transLetter2number) as a streamlined pipeline to manage these issues, after which quality control of the genotypic data is performed using the GSDataQC module. First, G2P applies the GSFiltering module to perform SNP filtration using the default criteria: missing rate > 0, MAF (minor allele frequency) < 0.05, and LD-based pruning to remove a potentially large amount of low-quality and excessive SNPs. Then, “GSTransForm” and “transLetter2number” are jointly run to transform the character-based genotypes (A, C, G, T) in binary PLINK format to numeric genotypes (0, 1, 2). Finally, GSDataQC examines the genotypic and phenotypic dataset either to remove samples or SNPs with too many missing values or to perform imputation.

In our analysis of the CUBIC data, after preprocessing of the raw genotypic and phenotypic data, a highly condensed marker set containing 9,286 SNPs and a table of summarized phenotypic values of the three traits were generated. This demo dataset is included in the G2P package, and can be called by using the “data (cubic)” function. To construct an independent testing set (the Test-dataset) for model validation, 404 lines were randomly selected, and the remaining 1,000 lines were used as the training set (the CV-dataset) for CV-based model evaluation.

**Preprocessing of real-world breeding data**

In our application of G2P to a real-world breeding data, after preprocessing of the raw genotypic of 5, 782 inbred lines and the 5 tester lines, 22, 599 high quality SNPs were retained for simulating the genotypic data of F_1_ hybrids. After generation of F1 genotypes, function “transLetter2number” from G2P were run to transform the character-based genotypes (A, C, G, T) to a numeric genotype format (0, 1, 2) for next model building. The raw phenotypic data of grain yield (GY) per unit collected in the two years and six locations planted in each of the ecological zones were processed by the BLUP algorithm. Phenotypic values of grain yield trait were normalized with Standard score method and obtained a normal distribution with mean equal to zero.

**References**

Bradbury, P. J., Zhang, Z., Kroon, D. E., Casstevens, T. M., Ramdoss, Y., and Buckler, E. S. (2007). TASSEL: software for association mapping of complex traits in diverse samples. *Bioinformatics* 23, 2633–2635. doi: 10.1093/bioinformatics/btm308

Browning, S. R., and Browning, B. L. (2007). Rapid and accurate haplotype phasing and missing-data inference for whole-genome association studies by use of localized haplotype clustering. *Am. J. Hum. Genet. 81, 1084–1097. doi: 10.1086/521987*

Danecek, P., Auton, A., Abecasis, G., Albers, C. A., Banks, E., DePristo, M. A., et al. (2011). The variant call format and VCFtools. *Bioinformatics* 27, 2156–2158. doi: 10.1093/bioinformatics/btr330

Purcell, S., Neale, B., Todd-Brown, K., Thomas, L., Ferreira, M. A., Bender, D., et al. (2007). PLINK: A Tool Set for Whole-Genome Association and Population-Based Linkage Analyses.Am. J. Hum. Genet. 81, 559–575. doi: 10.1086/519795

Yan, J., and Wang, X. (2022a). Machine learning bridges omics sciences and plant breeding. *Trends in Plant Science*. doi: 10.1016/j.tplants.2022.08.018.

Yan, J., and Wang, X. (2022b). Unsupervised and semi-supervised learning: the next frontier in machine learning for plant systems biology. *The Plant Journal* 111, 1527–1538. doi: 10.1111/tpj.15905.

Yang, J., Lee, S. H., Goddard, M. E., and Visscher, P. M. (2011). GCTA: A Tool for Genome-wide Complex Trait Analysis. *Am J Hum Genet* 88, 76–82. doi: 10.1016/j.ajhg.2010.11.011.

Zhou, X., and Stephens, M. (2012). Genome-wide efficient mixed-model analysis for association studies. Nat. Genet. 44, 821. doi: 10.1038/ng.2310
